# Statins Are Not Associated with Improved Bladder Cancer Outcomes in Patients with Early-Stage Bladder Cancer Treated with BCG Immunotherapy

**DOI:** 10.3390/cancers17122027

**Published:** 2025-06-17

**Authors:** Estelle Ndukwe, Paz Lotan, Michael Risk, Elizabeth L. Koehne, Daniel D. Shapiro, Robert P. Tyllo, Glenn O. Allen, E. Jason Abel, David F. Jarrard, Kyle A. Richards

**Affiliations:** 1 University of Wisconsin School of Medicine and Public Health, Madison, WI 53726, USA; 2 Department of Urology, University of Wisconsin Madison, Madison, WI 53792, USA; 3 Division of Urology, William S. Middleton Memorial Veterans Hospital, Madison, WI 53705, USA

**Keywords:** statins, BCG immunotherapy, NMIBC

## Abstract

**Background**: Statins are commonly used cholesterol-lowering drugs with evidence of additional chemoprotective and immunomodulatory effects resulting from the inhibition of DNA replication, cell proliferation, and TH1-cell inhibition. There are conflicting reports regarding the potential benefit of concurrent statin treatment on non-muscle invasive bladder cancer (NMIBC) and specifically on intravesical Bacillus Calmette–Guerin (BCG) outcomes. We therefore aimed to analyze the effects of concurrent BCG and statin use in patients with NMIBC. **Methods**: National Veterans Affairs databases were used to retrospectively identify men with NMIBC between 2000 and 2010 who were treated with BCG. Pharmacy data was interrogated, and patients were divided according to statin therapy status. Statins had to be given at the beginning of BCG treatments and continued for at least 6 months. Cox proportional hazard ratios after inverse propensity score-weighted and competing risks adjustments were calculated for recurrence, secondary events (e.g., progression), cancer-specific survival, and overall survival. **Results**: Among 8814 patients, with a median follow-up of 11.3 years, statins were used by 38% of the patients. Patients taking statins were older (71 vs. 68, *p* < 0.0001), had more comorbidities (Charlson Comorbidity Index (CCI > 2; 38.6% vs. 31.4%, *p* < 0.0001), and had a higher-grade disease (40.2% vs. 34.3%, *p* < 0.0001) compared to those not on statins. After adjusting for stage, grade, age, race, CCI, agent orange exposure, and year of diagnosis, Cox proportional hazard analysis revealed no association with recurrence (HR 1.05, 95% CI 0.97–1.15, *p* = 0.23), secondary events (HR 0.91, 95% CI 0.80–1.05, *p* = 0.189), or bladder cancer specific survival (HR 0.88, 95% CI 0.76–1.02, *p* = 0.09) of statin use. However, statins were associated with improved overall survival (HR 0.89, 95% CI 0.83–0.96, *p* = 0.002). **Conclusions**: Concurrent statin and BCG use in patients with NMIBC was associated with improved overall survival, but not recurrence, secondary events, or bladder cancer-specific survival. These results confirm the real-world well-established cardiovascular benefit of statin treatment and primary preventive care. However, this large population study did not find any association between statins and the outcomes of patients with NMIBC treated with BCG immunotherapy.

## Simple Summary

While BCG immunotherapy remains the gold standard of care for non-muscle invasive bladder cancer (NMIBC) with an intermediate or high risk of progression, many problems have come to light with its increasing clinical application, including its attainability, intolerance, and relapse odds. There are conflicting reports on the use of statins in addition to standard cancer treatment to improve outcomes for patients due to its inhibition of DNA replication, cell proliferation, and TH1 cell inhibition with various cancers in vitro. This study aims to examine the presence of any sort of relationship between BCG treatment, statins, and their potential concomitant effect on non-muscle invasive bladder cancer. If statins prove to be the miracle addition that some studies have claimed them to be, it could mean a breakthrough in cancer treatment options.

## 1. Introduction

Bacillus Calmette–Guerin (BCG) is the current first-line standard of care therapy for non-muscle invasive bladder cancer (NMIBC) at an intermediate or high risk of progression [1,2,3]. Despite this, many problems have arisen in the clinical application of BCG, including its availability, tolerance and toxicity, and efficacy for a subgroup of patients with particularly high-risk NMIBC. This leaves these patients with options that are either not as effective or much more invasive, such as radical cystectomy.

Statins are a commonly used cholesterol-lowering drug. However, since the early 1990s, studies have shown evidence for their additional chemoprotective and immunomodulatory effects resulting from the inhibition of DNA replication, cell proliferation, and TH1-cell inhibition [4,5,6,7,8,9,10]. Statins block the HMG-CoA reductase synthesis of mevalonate, an important and complex signaling pathway for the production of cholesterol, vitamin D, lipoproteins, polyol, and ubiquinone [11,12]. Evidence has pointed to the activation of the mevalonate (MVA) pathway in various cancers, aiding the adaptability and proliferation of tumor cells [13,14]. The MVA pathway uses acetyl-COA, NADPH, and ATP to produce strolls and isoprenoids that are essential for tumor growth. The transcription of genes encoding MVA pathway enzymes is controlled primarily by the sterol regulatory element-binding protein (SREBP) family. These MVA pathway enzymes have been shown to be essential in the survival of several cancer cell lines. When these enzymes are low in the setting of highly proliferative cancer cells needing to produce membranes rapidly, SREBP is activated to make more enzymes and the MVA pathway is ramped up. An increased demand for cholesterol for the growth and survival of cancer cells leaves way for a potential anticancer strategy. It is no wonder that this pathway continues to be studied and discussed in the conversation of possible therapeutic targets for various cancers and that statins continue to be implicated in this discussion. Although the cholesterol-lowering effects of statins are due to the inhibition of the MVA pathway in the liver, lipophilic statins (atorvastatin, simvastatin, and lovastatin) have shown some activity in extra-hepatic tissues, further demonstrating their possible use as an anticancer agent in various cancers. It is, however, unknown whether these statins accumulate in tumor tissues to be cytotoxic to cancer cells. Mechanistically, it has been proposed that the inhibition of the mevalonate (MVA) pathway synergizes with immune checkpoint blockades [15]. With statins’ direct effects on HMG-CoA reductase, thereby preventing further production of mevalonate, the rationale to study statins in patients receiving immunotherapy such as BCG for bladder cancer is warranted. Various studies have shown an increase in efficacy with statin use in combination with standard of care treatment for some cancers. Several in vitro experiments have elicited the cytotoxic effect of different statins on bladder cancer lines; Kamat et al. reported that atorvastatin inhibited cell proliferation and DNA synthesis in RT4 and KU-7 bladder cancer lines [16], Wang et al. reported simvastatin-induced cell cycle arrest in T24 and 5637 cell lines [17], and Parada et al. conducted studies that elicited atorvastatin’s inhibition of bladder cancer development via antiproliferative, anti-inflammatory, and antioxidant profiles [18]. In addition, statins have been implicated in their regulation of autophagy, ferroptosis, and proptosis in vitro, which can modulate the tumor microenvironment [19,20,21]. The precise mechanism of these processes is unknown, but the effect seems to be dependent on the inhibition of HMG-CoA reductase as mevalonate supplementation to the medium-blocked induction of autophagy. In a similar vein, various studies have shown that statins can directly and specifically trigger the apoptosis of tumor cells while leaving normal cells alone to retain their proliferative potential, therefore pointing to its possible activating role in the immune system [22,23,24,25].

While these studies could point to an association of improved cancer outcomes with statin use, there are conflicting reports regarding the potential benefit of concurrent statin treatment on NMIBC and specifically on intravesical BCG outcomes. One retrospective study published in the *New England Journal of Medicine* by Hoffmann et al. analyzed clinical outcomes for patients who had received BCG immunotherapy for the treatment of NMIBC, and found that 53% of the patients who took statins developed more aggressive tumors, whereas this change occurred in only 18% of the patients who did not take statins [26]. Another study conducted by Liu et al. found that patients with NMIBC who received BCG treatment with statin administration had significantly better overall survival, cancer-free specific survival, and progression-free survival [27]. The study also found that patients who had started statins before BCG treatment had better cancer-free specific survival and progression-free survival. And yet, another systematic review and meta-analysis study conducted by Symvoulidis et al. included 32 reports and demonstrated no association between statin administration and urinary bladder cancer local control, recurrence, survival, or mortality, or between statin administration and BCG immunotherapy effectiveness [28]. These are just a few representative studies to show that the consensus on statins’ effects on NMIBC is still up for debate.

This study aimed to analyze the effects of concurrent BCG and statin treatment on patients with NMIBC, as we hypothesized that there was no improvement in recurrence-free survival, overall survival, secondary events, and bladder cancer-specific survival with the use of statins and BCG immunotherapy for patients with NMIBC.

## 2. Materials and Methods

### 2.1. Data Source

This study was approved by the IRB and is the result of work supported by the facilities and resources at the William S. Middleton Memorial Veterans Hospital in Madison, Wisconsin. The Department of Veterans Affairs (VA) provides care to more than 9 million enrolled veterans and has over 1400 sites across the nation [29]. Since 1999, the VA has documented all aspects of patient care within its electronic health record (EHR) system [30]. This includes laboratory results, imaging reports, diagnoses, procedures, medications, provider notes, and other relevant clinical information. For this study, data were extracted from individual VA EHR systems and consolidated within the national VA Corporate Data Warehouse [31].

### 2.2. Study Population

The National VA databases were queried to produce a cohort. Initially, 117,402 patients diagnosed with bladder cancer (ICD-9 188) were identified from 1 January 2000 to 31 December 2010. Patients with stage T2 or greater were excluded. Longitudinal data was compiled until death or the end of the study on 31 December 2018, depending on whichever took place first. Pharmacy data was interrogated, and patients were divided according to statin therapy status.

### 2.3. Outcomes of Interest

The primary outcome of this study was recurrence-free survival (RFS). Secondary outcomes included secondary events (SEs), bladder cancer-specific survival (BCSS), and overall survival (OS). OS was defined as the time from bladder cancer diagnosis to death from any cause, or censoring at the end of the study period. SEs were defined as the first occurrence of cystectomy, radiation therapy, systemic chemotherapy, or death from bladder cancer due to disease progression or treatment-refractory disease. Recurrence was defined as any transurethral resection of bladder tumor (TURBT), confirmed via histologic analysis, occurring within less than 3 months after an office cystoscopy at any point following initial diagnosis. This criterion was designed to exclude planned repeat TURBTs from being misclassified as recurrence. The time interval from initial diagnosis to death (from any cause or from bladder cancer), SE, or recurrence was used as the dependent variable in all survival analyses. Covariates included demographic and clinical factors such as age at diagnosis, race, Charlson Comorbidity Index (CCI), year of diagnosis, tumor stage, and grade, which were used for adjustment in multivariable models.

### 2.4. Predictors and Measures

The statin group was defined as any veteran with documented statin use from the beginning of BCG treatment and continued for at least 6 months based on pharmacy refill data and patient endorsement. Cox proportional hazard ratios were analyzed after inverse propensity score-weighted and competing risks adjustments were calculated for recurrence, secondary events, cancer-specific survival, and overall survival.

### 2.5. Propensity Score Adjustment

Propensity scores were estimated using a multinomial logistic regression model, with statin use specified as the categorical outcome variable. Predictor variables included age at diagnosis, race, Charlson Comorbidity Index (CCI), year of diagnosis, tumor stage, and tumor grade. These propensity scores were subsequently applied as inverse probability of treatment weights in an inverse propensity score weighting (IPSW) approach to adjust for baseline differences between the statin-exposed and unexposed groups. This method was used to balance covariates in the final Cox proportional hazards model evaluating survival outcomes. This method was preferred over matching to account for the multiple variable models and avoid arbitrary matching decisions. It was also the better analysis method for this larger study.

### 2.6. Statistical Analysis

All statistical analyses were conducted using Stata version 16. Continuous variables were compared using the Mann–Whitney U test, while categorical variables were analyzed using the chi-squared test. Baseline demographic and clinical characteristics were evaluated by treatment group as presented in the corresponding tables. Following inverse propensity score weighting (IPSW) was used to account for potential confounding, multivariable Cox proportional hazards regression models were employed to examine the association between statin exposure and outcomes, including recurrence-free survival (RFS), secondary events (SEs), and overall survival (OS). All statistical tests were two-sided, with *p*-values < 0.05 considered statistically significant.

## 3. Results

In the end, this study consisted of 8814 patients with NMIBC that were diagnosed between 2000 and 2010 and treated with BCG. The mean follow-up time was 11.3 years. A total of 3333 patients (37.8%) had a statin prescription during BCG treatment. There were significant baseline differences between the statin and non-statin cohorts in age, stage, grade, and CCI (Table 1). Patients taking statins were older (71 vs. 68, *p* < 0.0001), had more comorbidities (Charlson Comorbidity Index (CCI > 2; 38.6% vs. 31.4%, *p* < 0.0001), and had a higher-grade disease (40.2% vs. 34.3%, *p* < 0.0001) compared to those not on statins.

After adjusting for stage, grade, age, race, CCI, and year of diagnosis, there was no association of statin use with RFS (Table 2, red box). Statin use was also not associated with the other SEs of interest (Table 3, red box) or BCCS (Table 4, red box). However, statin use was associated with improved OS (Table 5, red box).

Even after adjusting for possible confounders such as stage, grade, age, race, CCI, and year of diagnosis, statins were still associated with improved overall survival. Other confounding variables include healthcare utilization bias (since those actively on a statin might be more health conscious or have better access to care), measurement bias (since pharmacy fill data was used to determine stain exposure and this may not reflect actual adherence or dosage intensity), and other non-cancer causes of death. Barring that, given this study’s large cohort and extended median follow-up time of 11.3 years, this study was well-powered to detect clinically meaningful differences in bladder cancer-related outcomes between statin users and non-statin users. The observed number of events across outcomes—recurrence, progression, cancer specific death, and overall mortality—provided sufficient power for time to event analyses using Cox proportional hazards modeling. The lack of statistically significant associations between statin use and bladder cancer-specific outcomes (e.g., recurrence, secondary events, and cancer-specific survival) suggests that, if a true effect exists, it is likely to be modest. Furthermore, the power to detect smaller effect sizes may have been limited by variability in treatment exposure, potential residual confounding, and outcome heterogeneity despite adjustment via inverse propensity score weighting.

## 4. Discussion

In this retrospective observational study, we evaluated the potential association between statin use and oncologic outcomes in patients with non-muscle invasive bladder cancer (NMIBC) treated with intravesical Bacillus Calmette–Guérin (BCG). To our knowledge, this represents the largest observational analysis to date examining the possible interaction between statin therapy and clinical outcomes in this patient population. Among the 8814 patients analyzed, statin use was not independently associated with improved recurrence-free survival, secondary event-free survival, or bladder cancer-specific survival. However, statin exposure was associated with improved overall survival, suggesting a potential benefit unrelated to bladder cancer-specific progression. As mentioned previously, prior research into the effect of concomitant statin use in patients with bladder cancer has yielded conflicting results. A systematic review conducted by Symvoulidis et al. evaluated 32 studies that investigated the effects of statin therapy on bladder cancer [28]. Their analysis of these studies showed no association between statin administration and bladder cancer survival or mortality, or between statin administration and BCG immunotherapy effectiveness. More specifically, Symvoulidis’ review identified six studies that investigated the possible effects of statins on BCG immunotherapy. Notably, the stages of cancer that each study analyzed in this systematic review were not identified and there was no exclusion criteria based on stage for this article.

One of the studies conducted by Hoffmann et al. in 2006 found that the discontinuation of statins during BCG therapy might be beneficial for patients due to the study’s reported higher rate of tumor progression in statin users [26]. This study included a cohort of 84 patients, with 19 of them being statin users. Of those statin users, 53% developed more aggressive tumors, whereas this change only occurred in 18% of patients who did not take statins. And while these findings are somewhat distinctive, the small sample size and failure to specify the stage and grade of tumors that were included for analysis point to significant limitations on the generalizability of this study.

Another study carried out by Kamat et al. in 2007 included a cohort of 156 patients receiving BCG, and found no effect of statin use on recurrence, progression, or number of deaths during BCG therapy [16]. This study was conducted in direct response to the Hoffmann study and their conclusion that the discontinuation of statin therapy during BCG immunotherapy might improve the clinical outcome. And yet, another study conducted by Berglund et al. in 2008, which included a cohort of 952 patients, concluded the same as the Kamat study [32]. The three remaining relevant studies included in the systematic review had significantly smaller cohort numbers and found no association between statin use and improved bladder cancer survival.

A study with a comparable scope of patients and median follow-up time (11 years) published by Hong Kong-based Liu et al. in September of 2024 concluded that statin treatment appears to offer a protective effect on overall survival and cancer-specific survival for patients with NMIBC receiving adjuvant intravesical BCG [27]. However, while their original cohort included 1163 patients with NMIBC who received BCG therapy and were also using a statin, within that group, only 637 patients had adequate BCG treatment. In addition, some important covariates, like tumor staging, were not used assessed due to an inability to retrieve those variables from the electronic database.

Our study suggests that patients with NMIBC receiving BCG therapy did not have better outcomes when treated simultaneously with statins. The evidence from this study suggests that statins are not a necessary additional treatment strategy for urologists to adopt for their patients with NMIBC. This conclusion is not meant to encourage patients currently on BCG immunotherapy for NMIBC with concurrent statin use to do away with their prescription during treatment for bladder cancer. These results suggest the already well-established cardiovascular benefits of statin treatment and its role in preventive care. Patients should be encouraged to continue their appropriate care as directed by other primary care/cardiac providers, since non-urologic issues can have just as much or more of an effect on longevity.

Nonetheless, we recognize the limitations of this study. First, due to the nature of our cohort (NMIBC receiving BCG), the results may not be generalizable to all bladder cancer patients, such as those with advanced disease. Furthermore, the type of statin used was not elucidated during our study, even though some statins have been shown to have a higher proapoptotic activity than others during concomitant cancer treatment [11]. Our recurrence and SE endpoints were measured systematically using proxies and administrative data, but if anything, they were likely overestimated, as those points were unable to be confirmed by tissue pathology. Despite this, they were measured uniformly in both statin users and non-users. Finally, while the use of inverse propensity score weighting (IPSW) and multivariable regression analyses helped to mitigate selection bias and account for measured confounders, the possibility of residual unmeasured confounding cannot be excluded. A major strength of this study lies in its large sample size and extended duration of follow-up, which enhances the robustness and generalizability of the findings.

## 5. Conclusions

Concurrent statin and BCG use in patients with NMIBC was associated with improved overall survival, but not RFS, SEs, or BCSS. These results suggest that statins provide an overall cardiovascular benefit to patients that need them, but that they lack an effect on the oncologic outcomes of NMIBC patients treated with BCG immunotherapy.

Though statins’ in vitro mechanisms and efficacy cannot be denied, these effects may not be easily translated clinically. Various reasons can account for this discrepancy, including tumor microenvironment heterogeneity across individual patients, cancer cell line homogeneity, drug pharmacokinetics and pharmacodynamics, and a lack of host immune response in these in vitro studies.

## Figures and Tables

**Table 1:** Baseline demographic and clinical characteristics of the cohort of patients with bladder cancer treated with BCG.

	All	Statin	Non-Statin	*p*-Value
No. of patients (%)	8814 (100)	3333 (37.8)	5481 (62.2)	
Age, median (IQR)	69.0 (62.0–77.0)	71.0 (64.0–78.0)	68.0 (60.0–76.0)	<0.0001
Sex, male, n (%)	8769 (99.2)	3325 (99.4)	5444 (99.1)	0.149
Race, n (%)				0.120
White	6995 (79.1)	2713 (81.1)	4282 (77.9)	
Black	612 (6.9)	208 (6.2)	404 (7.4)	
Other	1233 (13.9)	191 (12.7)	808 (14.7)	
Charlson Comorbidity index (CCI), n (%)				<0.0001
0–1	4614 (65.3)	1932 (61.5)	2682 (68.5)	
2–3	1878 (26.6)	898 (28.5)	980 (25.0)	
>3	570 (8.1)	319 (10.1)	251 (6.4)	
Year of diagnosis (%)				<0.0001
2000–2005	3326 (37.6)	1030 (30.8)	2296 (41.8)	
2006–2010	4374 (49.2)	2171 (64.9)	2176 (39.6)	
Other Year	1167 (13.2)	145 (4.3)	1022 (18.6)	
Stage (%)				0.0125
Ta	3335 (44.3)	1358 (45.1)	1977 (43.8)	
CIS	727 (9.7)	321 (10.7)	406 (9.0)	
T1	3462 (46.0)	1333 (44.3)	2129 (47.2)	
Grade, high, n (%)	3227 (36.5)	1345 (40.2)	1882 (34.3)	<0.0001
Recurrence, n (%)	5817 (65.8)	2283 (68.2)	3534 (64.3)	0.0002
Time to recurrence, months median (IQR)	6.0 (1.2–22.3)	3.5 (1.0–12.1)	8.0 (1.3–35.8)	<0.0001
Death from bladder cancer, n (%)	106.7 (59.4–145.0)	102.3 (59.9–130.7)	110.4 (59.2–157.8)	<0.0001
Time to death from bladder cancer, months median (IQR)	1896 (21.5)	686 (20.5)	1210 (22.0)	0.090
Follow-up time, months, median (IQR) *	33.7 (13.0–77.1)	31.4 (13.0–64.8)	35.7 (12.9–86.2)	<0.0001

CCI: Charleston Comorbidity Index, * follow-up time for those that did not die.

**Table 2:** Cox proportional hazards multivariable analysis assessing predictors of recurrence after inverse propensity score-weighted (IPSW) and competing risks adjustments.

	Hazard Ratio	95% CI	*p*-Value
Statin History for Patients			
No Statin Rxs	Referent	Referent	
Statin Rx	1.05	0.95–1.16	0.31
Stage			
Ta	Referent	Referent	
CIS	0.92	0.73–1.15	0.46
T1	0.91	0.82–0.01	0.07
Grade			
Low	Referent	Referent	
High	0.93	0.84–1.03	0.15
Age (continuous)	1.01	1.00–1.02	<0.0001
Race			
White	Referent	Referent	
Black	0.88	0.72–1.06	0.19
Other	0.92	0.76–1.10	0.35
Charlson Comorbidity index			
≤1	Referent	Referent	
2–3	0.85	0.75–0.95	0.004
>3	0.71	0.58–0.87	0.001
Year of diagnosis			
2000–2005	Referent	Referent	
2006–2010	0.89	0.78–1.05	0.12

**Table 3:** Cox proportional hazards multivariable analysis assessing predictors of secondary events after inverse propensity score-weighted (IPSW) and competing risks adjustments.

	Hazard Ratio	95% CI	*p*-Value
Statin History for Patients			
No Statin Rxs	Referent	Referent	
Statin Rx	0.91	0.80–1.05	0.189
Stage			
Ta	Referent	Referent	
CIS	1.31	0.96–1.77	0.09
T1	1.53	1.33–1.77	<0.0001
Grade			
Low	Referent	Referent	
High	1.40	1.22–1.61	<0.0001
Age (continuous)			
Race			
White	Referent	Referent	
Black	1.29	1.02–1.63	0.03
Other	0.98	0.74–1.29	0.87
Charlson Comorbidity index			
≤1	Referent	Referent	
2–3	1.00	0.86–1.17	0.95
>3	1.12	0.86–1.46	0.40
Year of diagnosis			
2000–2005	Referent	Referent	
2006–2010	1.08	0.93–1.25	0.29

**Table 4:** Cox proportional hazards multivariable analysis assessing predictors of bladder cancer-specific survival after inverse propensity score-weighted (IPSW) and competing risks adjustments.

	Hazard Ratio	95% CI	*p*-Value
Statin History for Patients			
No Statin Rxs	Referent	Referent	
Statin Rx	0.88	0.76–1.02	0.09
Stage			
Ta	Referent	Referent	
CIS	1.09	0.76–1.55	0.65
T1	1.45	1.24–1.70	<0.0001
Grade			
Low	Referent	Referent	
High	1.40	1.20–1.64	<0.0001
Age (continuous)	1.04	1.03–1.05	<0.0001
Race			
White	Referent	Referent	
Black	1.46	1.15–1.86	0.002
Other	1.16	0.89–1.51	0.28
Charlson Comorbidity index			
≤1	Referent	Referent	
2–3	1.13	0.96–1.34	0.15
>3	1.86	1.45–2.38	<0.0001
Year of diagnosis			
2000–2005	Referent	Referent	
2006–2010	0.88	0.75–1.03	0.10

**Table 5:** Cox proportional hazards multivariable analysis assessing predictors of overall survival after inverse propensity score-weighted (IPSW) and competing risks adjustments.

	Hazard Ratio	95% CI	*p*-Value
Statin History for Patients			
No Statin Rxs	Referent	Referent	
Statin Rx	0.89	0.83–0.96	0.002
Stage			
Ta	Referent	Referent	
CIS	1.13	0.96–1.33	0.14
T1	1.17	1.08–1.26	<0.0001
Grade			
Low	Referent	Referent	
High	1.27	1.17–1.37	<0.0001
Age (continuous)	1.06	1.06–1.07	<0.0001
Race			
White	Referent	Referent	
Black	0.97	0.84–1.13	0.71
Other	0.94	0.81–1.10	0.44
Charlson Comorbidity index			
≤1	Referent	Referent	
2–3	1.28	1.18–1.40	<0.0001
>3	2.14	1.88–2.43	<0.0001
Year of diagnosis			
2000–2005	Referent	Referent	
2006–2010	1.09	1.01–1.19	0.03

**Figure A1:**
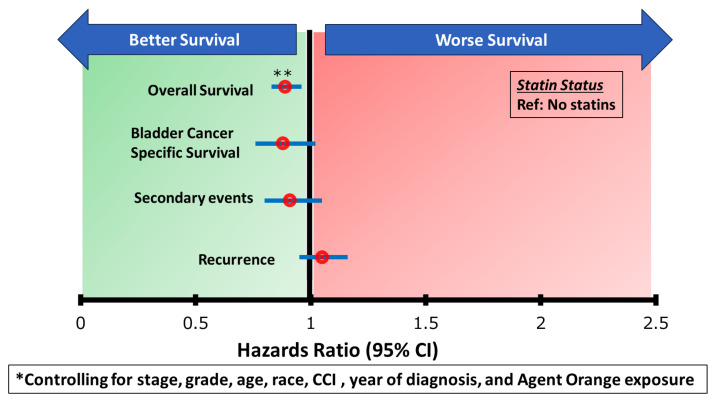
Multivarible Cox Proportional Hazards Analysis.

See Figure A1 in Appendix A for a graphical representation of the multivariable cox proportional hazards analysis performed for each parameter.

## Data Availability

Data is not available due to privacy restrictions.
